# Integrated Evaluation of Alkaline Tolerance in Soybean: Linking Germplasm Screening with Physiological, Biochemical, and Molecular Responses

**DOI:** 10.3390/plants15020222

**Published:** 2026-01-10

**Authors:** Yongguo Xue, Zichun Wei, Chengbo Zhang, Yudan Wang, Dan Cao, Xiaofei Tang, Yubo Yao, Wenjin He, Chao Chen, Zaib_un Nisa, Xinlei Liu

**Affiliations:** 1 Soybean Research Institute, Heilongjiang Academy of Agricultural Sciences, Harbin 150086, China; xyg81@126.com (Y.X.); caodan825@163.com (D.C.); yaoyubo2009@aliyun.com (Y.Y.); nkydds@126.com (W.H.); 2 Department of Chemistry and Molecular Biology, School of Life Science and Technology, Harbin Normal University, Harbin 150025, China; wzcfyywzy@163.com (Z.W.); zcblss@126.com (C.Z.); wangyudan0662@163.com (Y.W.); chchao@hrbnu.edu.cn (C.C.); 3 General Botany Laboratory, Institute of Molecular Biology and Biotechnology, The University of Lahore, Defence Road Campus, Lahore 54000, Pakistan

**Keywords:** soybean, alkali tolerance, transcriptome, physiological mechanism

## Abstract

Soybean (*Glycine max* L.) is an essential food and economic crop in China, yet its growth and yield are severely constrained by saline–alkali stress. A saline–alkali soil exacerbates root absorption barriers, leading to 30–50% yield losses. Understanding the mechanisms underlying alkali tolerance is therefore crucial for developing stress-resilient soybean varieties and improving the productivity of saline–alkali land. In our previous study, we evaluated 99 soybean germplasms from Northeast China and obtained the alkali-tolerant varieties HN48 and HN69, along with the alkali-sensitive varieties HNWD4 and HN83. In this study, fifteen-day-old soybean seedlings were subjected to (30 mM NaHCO_3_) alkali stress for 72 h, and whole plants were sampled to assess their morphology and physiology, while leaf tissues were harvested for biochemical analysis. For transcriptomic analysis, soybean seedlings were exposed to alkali stress (50 mM NaHCO_3_, pH 9.0) for 6 h, and leaf and root tissues were harvested for RNA sequencing. The results showed that alkali-tolerant varieties mitigated these effects by suppressing excessive ROS generation by 55–63%, decreasing malondialdehyde (MDA) accumulation by 37–39%, and increasing photosynthetic efficiency by 18.3%, as well as accumulating more osmoprotectants and activating antioxidant enzymes such as superoxide dismutase (SOD) and catalase (CAT) under alkaline stress. Transcriptome analysis showed that the alkali-tolerant variety HN69 exhibited cultivar-specific enrichment of metabolism cytochrome P450, estrogen signaling, and GnRH signaling pathways under alkali stress. These results collectively indicate that alkali-tolerant soybean varieties adapt to alkali stress through coordinated multi-pathway responses, with differential pathway enrichment potentially underlying the variation in alkali tolerance between cultivars. Overall, this study elucidates the physiological and molecular mechanisms of alkali tolerance in soybean, providing a theoretical foundation for breeding stress-tolerant germplasms.

## 1. Introduction

Soil salinization and alkalization represent major abiotic stress factors that severely restrict global crop productivity and pose an escalating threat to food security. According to the second national soil survey, China possesses approximately 37 million hectares of usable saline–alkali soil resources, with the western Songliao Plain being identified as a major saline–alkali hotspot encompassing about 16.47 million hectares [1]. These soils are characterized by strong alkalinity and a high Na^+^ concentration, frequently causing sharp reductions in soybean yield of 30–50%. Soybean (*Glycine max* L.), an essential source of food, vegetable oil, and animal feed, remains in short domestic supply. In 2021, China’s soybean production reached only 16.4 million tons, while annual consumption exceeded 100 million tons, revealing a significant production–consumption gap [2]. Addressing this imbalance requires not only agronomic improvements but also genetic innovations that enhance crops’ resilience to environmental stresses. Therefore, it is imperative to identify and utilize alkali-tolerant genetic resources within indigenous soybean germplasms and elucidate the underlying molecular and physiological mechanisms of alkali stress adaptation [3]. Such insights will provide a scientific foundation for breeding stress-resilient varieties, expanding soybean cultivation beyond its current geographic limits, and strengthening national self-sufficiency in soybean production [4].

Alkali stress triggers multi-dimensional physiological responses in plants. Morphologically, it inhibits cell division in the root apical meristem [5], restricts primary root elongation (shortening by 20–40%), and significantly reduces lateral root initiation frequency (>30%), accompanied by leaf chlorosis and plant dwarfing [6]. Damage to subcellular organelles is another defining symptom [7], characterized by thylakoid swelling within chloroplasts and the disintegration of mitochondrial cri. These structural alterations severely hinder photophosphorylation and compromise overall energy metabolism. At the physiological level, metabolic imbalances are evident through a sharp rise in reactive oxygen species (ROS), which induces elevated levels of malondialdehyde (MDA), a key indicator of lipid peroxidation, and results in disrupted membrane stability, reflected by a rise in electrolyte leakage [8,9]. As a result, photosynthetic organ function declines, evidenced by accelerated chlorophyll degradation, reduced maximum quantum efficiency of photosystem II (PSII, Fv/Fm), and a significant drop in net CO_2_ assimilation rate [10,11].

To cope with alkali stress, soybean has evolved coordinated multi-level defense strategies [12]. First, the antioxidant system is activated, significantly upregulating the activity of key enzymes like superoxide dismutase (SOD) and peroxidase (POD) to effectively scavenge excess ROS [13,14]. Second, osmotic regulators (e.g., proline, soluble sugars) are synthesized abundantly, with accumulation levels increasing 2-3-fold, maintaining cellular hydration status through osmotic potential adjustment. Third, ion homeostasis is primarily maintained via the Salt Overly Sensitive (SOS) signaling pathway. This pathway drives cytoplasmic Na^+^ efflux and vacuolar sequestration, thereby ensuring dynamic K^+^/Na^+^ balance [15]. Notably, alkali-tolerant genotypes typically maintain a higher K^+^/Na^+^ ratio than sensitive ones [16]. Nevertheless, the identification of alkali-tolerant soybean varieties and the elucidation of their physiological and molecular mechanisms of alkali tolerance are still pressing issues in soybean production.

Considerable genetic variation in alkali tolerance exists among soybean germplasms, as previous screenings of over 140 accessions have identified several tolerant lines. However, most studies focus narrowly on the germination stage and lack standardized evaluation systems. To address these gaps, in our previous study, we evaluated 99 soybean germplasms from Northeast China and obtained the alkali-tolerant varieties HN48 and HN69, along with the alkali-sensitive varieties HNWD4 and HN83. We hypothesized that alkali-tolerant soybean varieties (HN48 and HN69) and alkali-sensitive varieties (HNWD4 and HN83) exhibited differences in physiological, biochemical, and antioxidant enzyme activity profiles, as well as in their regulatory gene expression under alkali stress. Therefore, we elucidated the mechanisms of alkali-tolerant and alkali-sensitive varieties in response to alkaline stress by integrating phenotypic, biochemical, and molecular analyses.

## 2. Results

### 2.1. Assessment of Morphological Alterations Under Alkali Stress

**Figure 1:**
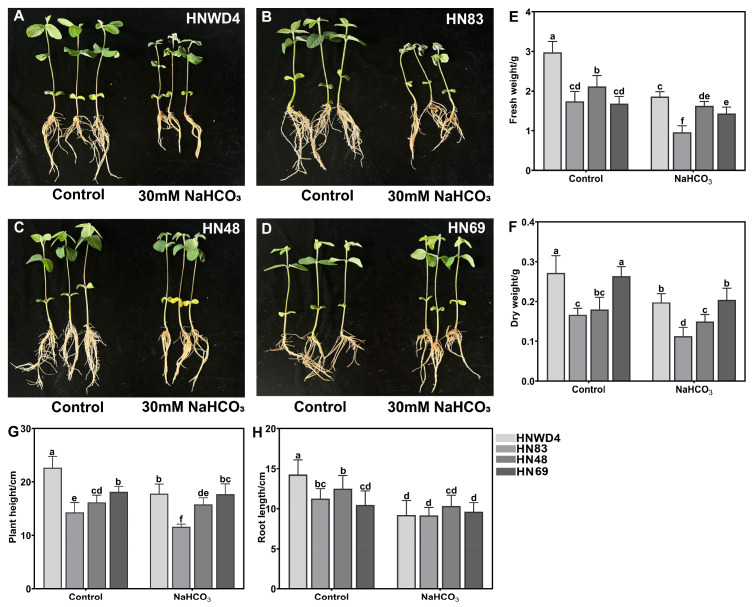
Phenotypic responses of soybean seedlings to alkaline stress. (**A**–**D**) Phenotypic analysis of soybean seedlings (HN48, HN69, HN83, and HNWD4) under alkaline stress. Seedlings were grown hydroponically in Hoagland’s nutrient solution, with the control group or the 30 mM NaHCO_3_ treatment group. Quantitative analysis of key growth parameters, including plant fresh weight (**E**), dry weight (**F**)**,** plant height (**G**), and root length (**H**), in soybean accessions subjected to 30 mM NaHCO_3_, compared with control conditions. Different lowercase letters above the bars indicate significant differences. Significant differences between control and treatment groups were determined using one-way ANOVA, followed by Duncan’s multiple range test (*p* < 0.05).

To evaluate the morphological responses of soybean seedlings (HN48, HN69, HN83, and HNWD4) to alkaline stress, *Glycine max* accessions were cultivated hydroponically in Hoagland’s nutrient solution. The treatment group was exposed to 30 mM NaHCO_3_. The results showed that alkaline stress markedly inhibited seedling growth compared to the control, as evidenced by pronounced wilting symptoms and a substantial reduction in overall biomass accumulation (Figure 1A,B). Across all tested accessions, exposure to alkaline conditions significantly decreased all key morphological parameters, including plant height, root length, fresh weight, and dry weight (Figure 1E–H). In particular, HNWD4 exhibited a 55.6% reduction in root length and a 66.7% decline in fresh weight, whereas HN83 showed a 44.4% reduction in fresh biomass (Figure 1E,H). In contrast, the more tolerant accessions maintained better morphological stability under stress. HN48 displayed no significant difference in plant height or dry weight relative to the control, while HN69 sustained comparable values for both height and root elongation (Figure 1G,H). Overall, the results demonstrate clear accession-specific variations in seedling morphogenesis under alkaline stress, with a strong positive correlation between morphological performance and genotypic tolerance capacity.

### 2.2. Measurement of Physiological Attributes in Soybean Seedlings Under Alkali Stress

**Figure 2:**
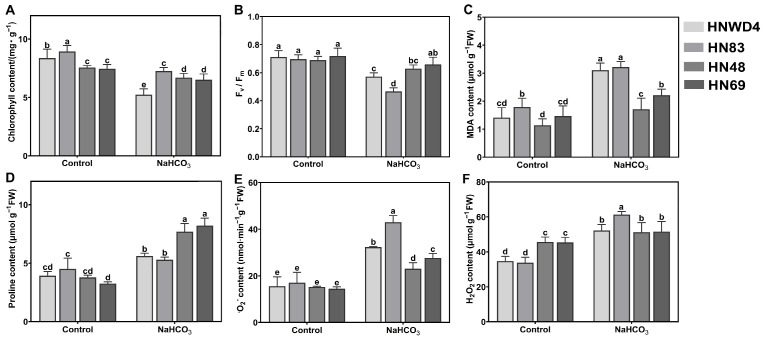
Determination of physiological indices in soybean seedlings under alkaline stress. Panels represent (**A**) chlorophyll content, (**B**) Fv/Fm, (**C**) MDA content, (**D**) proline content, (**E**) superoxide anion (O_2_^−^) production, and (**F**) hydrogen peroxide (H_2_O_2_) accumulation in different soybean varieties exposed to 30 mM NaHCO_3_ compared with controls. Data are expressed as mean ± standard error. Different lowercase letters above the bars indicate significant differences (*p* < 0.05), while identical letters denote no statistically significant differences.

Chlorophyll content analysis provides critical insights into how alkaline stress influences photosynthetic performance in soybean seedlings. As shown in Figure 2A, exposure to alkaline conditions led to a notable decline in chlorophyll content across different soybean accessions, with the extent of reduction varying by genotype. The alkali-sensitive varieties, HNWD4 and HN83, exhibited significant decreases of 38.8% and 16.6%, respectively, compared with the control. In contrast, the alkali-tolerant varieties, HN69 and HN48, experienced only modest reductions of 11.4% and 7.14%, respectively, suggesting a stronger capacity to preserve chlorophyll and sustain photosynthetic activity under stress. Chlorophyll fluorescence measurements further elucidated the physiological effects of alkaline stress on the photosynthetic apparatus. Assessment of the maximum photochemical quantum yield of PSII (Fv/Fm) revealed a marked decline in alkali-sensitive accessions, particularly in HNWD4 and HN83 (Figure 2B). In contrast, the tolerant variety HN69 exhibited only a minor reduction in Fv/Fm, maintaining near-normal photosynthetic efficiency. Overall, these findings indicate that alkali-tolerant soybean genotypes mitigate stress-induced damage by maintaining higher chlorophyll content, thereby enhancing their photosynthetic resilience under alkaline conditions.

### 2.3. Measurement of Stress Indicators (MDA, H_2_O_2_, O_2_^−^, and Proline Contents)

To elucidate the physiological responses of soybean seedlings to alkaline stress, changes in malondialdehyde (MDA) and hydrogen peroxide (H_2_O_2_) levels were quantified in leaf tissues. As shown in Figure 2C, the MDA content increased across all tested varieties under alkaline conditions, reflecting enhanced lipid peroxidation. The alkali-sensitive cultivars, HNWD4 and HN83, exhibited pronounced increases of 1.21-fold and 80%, respectively, whereas the alkali-tolerant cultivars, HN48 and HN 69, showed smaller increments of 38.9% and 37.5%, suggesting reduced oxidative damage and better membrane stability in the tolerant genotypes. As presented in Figure 2D, proline accumulation in tolerant varieties (HN48: 150.1%; HN69: 108.3%) was higher than that in sensitive varieties (40.7% and 10.6%). We hypothesized that the elevated proline content may play a role in alleviating alkali stress. Figure 2E illustrates that superoxide anion (O_2_^−^) production increased by 115.2–146.5% in the sensitive cultivars, compared to a more moderate rise of 52.1–90.6% in the tolerant cultivars. Similarly, Figure 2F shows that H_2_O_2_ levels rose sharply in sensitive varieties (50.3–81.2%), whereas tolerant varieties maintained relatively stable levels, with only slight increases (12.3–13.5%). Collectively, these results demonstrated that alkali-tolerant soybean varieties possess stronger antioxidant defense mechanisms, characterized by lower accumulation of MDA and reactive oxygen species (ROS). This physiological resilience effectively minimized the membrane lipid peroxidation and contributed to improved stress tolerance under alkaline conditions.

### 2.4. Determination of Antioxidant Enzyme Activities of Soybean Seedlings (HN48, HN69, HN83, and HNWD4) Under Alkaline Stress

**Figure 3:**
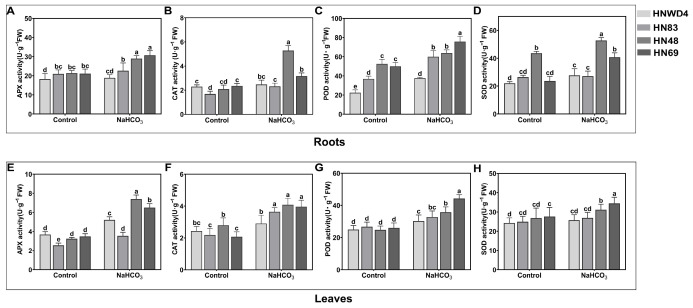
Changes in antioxidant enzyme activities in soybean leaves and roots under alkali stress; the enzyme activities of (**A**) APX, (**B**) SOD, (**C**) POD, and (**D**) CAT were detected in roots. The enzyme activities of (**E**) APX, (**F**) SOD, (**G**) POD, and (**H**) CAT were detected in leaves. Different lowercase letters above the bars indicate significant differences (*p* < 0.05), while the same letters denote no statistically significant differences.

Alkaline stress triggers the activation of the plant antioxidant defense system, in which changes in the activities of key enzymes, POD, CAT, SOD, and APX serve as important physiological markers of stress adaptation. To examine the differential antioxidant responses among soybean genotypes with varying levels of alkali tolerance, the activities of these enzymes were measured in the leaves and roots of four cultivars: HNWD4, HN83, HN48, and HN69. As shown in Figure 3A–H, alkali stress markedly influenced the antioxidant enzyme activities in soybean leaves. The tolerant cultivar HN48 exhibited a significant increase in APX activity (89.2% above the control), while the sensitive cultivar HNWD4 displayed only slight, non-significant increases in CAT and SOD activities (8.1% and 8.69%, respectively). In the roots, the tolerant cultivar HN69 demonstrated substantial enhancements in SOD and APX activities (75% and 50%, respectively), which were significantly higher than those observed in the sensitive cultivars. Overall, these findings suggest that alkali-tolerant soybean varieties alleviate oxidative damage by antioxidant enzymes, particularly POD and APX, in both leaves and roots. This enhanced enzymatic defense likely represents a key physiological mechanism underlying their superior tolerance to alkaline stress.

### 2.5. Differential Transcriptional Analysis of Soybean Seedlings (HN69 and HN83) Under Alkaline Stress

To further explore the difference between alkali-tolerant and alkali-sensitive soybean, we detected the transcriptome data of the alkali-tolerant HN69 and the alkali-sensitive HN83 under alkaline stress. Table S1 provides a summary of transcriptome sequencing data statistics for soybean cultivars under control and alkaline stress conditions. The table presents raw and clean read numbers, quality metrics (Q20 and Q30), clean-to-raw read ratios, and genome alignment rates for alkali-tolerant (HN69) and alkali-sensitive (HN83) soybean cultivars. Each treatment included three biological replicates. High-quality sequencing and alignment rates (>90%) confirm the reliability and suitability of the datasets for subsequent transcriptomic analyses.

**Figure 4:**
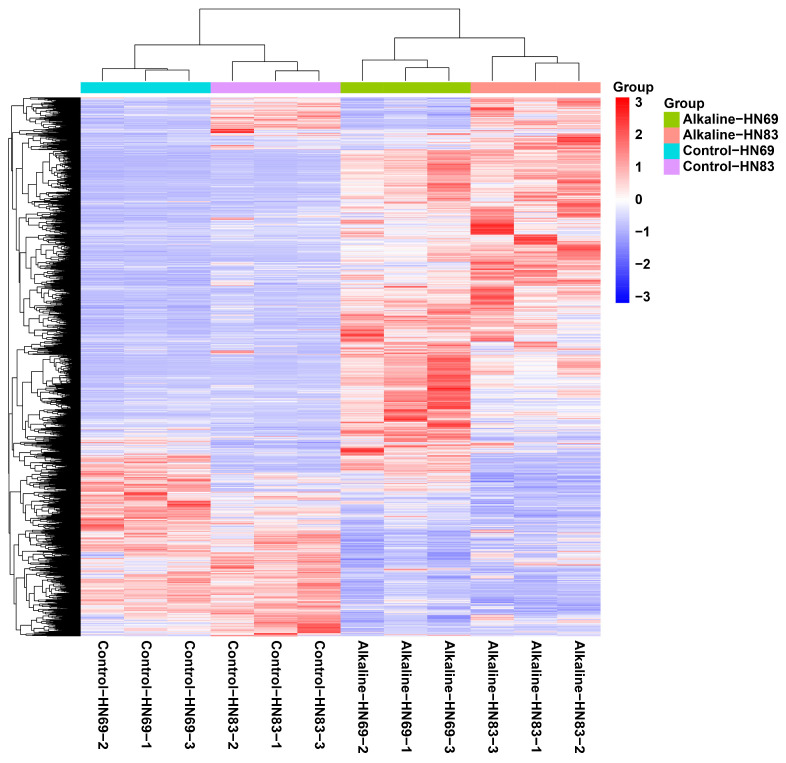
Cluster plot of the differentially expressed genes.

Differentially expressed genes (DEGs) were identified using a threshold of |log_2_ (fold change)| > 1. We found that 4134 DEGs were differentially expressed jointly between Alkaline-HN69 vs. Control-HN69 (3247 upregulated and 887 downregulated), 3324 genes were specifically expressed in the Alkaline-HN83 vs. Control-HN83 (3247 upregulated and 887 downregulated) group, and 1353 genes were specifically expressed in the Alkaline-HN69 vs. Alkaline-HN83 group (495 upregulated and 859 downregulated). Heatmap analysis clustered DEGs across groups (Figure 4). Samples or genes with shorter branch distances showed more similar expression patterns. The key findings were clear separation between HN69 and HN83 under both control and alkali stress conditions, indicating alkali-induced transcriptional reprogramming.

### 2.5.1. Functional Annotation and Enrichment Analysis of Differentially Expressed Genes

Hypergeometric testing using R’s phyper function (FDR-adjusted Q ≤ 0.05) identified statistically significant enrichment profiles. Upregulated DEGs in HN 69 were significantly enriched in biosynthesis, metabolism, and organic substance biosynthesis terms (Figure S1), while HN83 showed major enrichment in biosynthesis, metabolism, and biological processes (Figure S2), both indicating critical functional roles in alkali stress response.

KEGG pathway enrichment analysis (Q ≤ 0.05) further revealed that secondary metabolite biosynthesis and plant hormone signal transduction pathways contained the highest DEG numbers in both cultivars (Figures S3 and S4). Notably, HN 69 uniquely activated the metabolism-cytochrome P450, estrogen signaling, and GnRH signaling pathways. These results collectively indicate that soybean adapts to alkali stress through coordinated multi-pathway responses, with differential pathway enrichment potentially underlying the variation in alkali tolerance between cultivars.

### 2.5.2. Gene Expression Analysis Using qRT-PCR (Quantitative Real-Time PCR)

**Figure 5:**
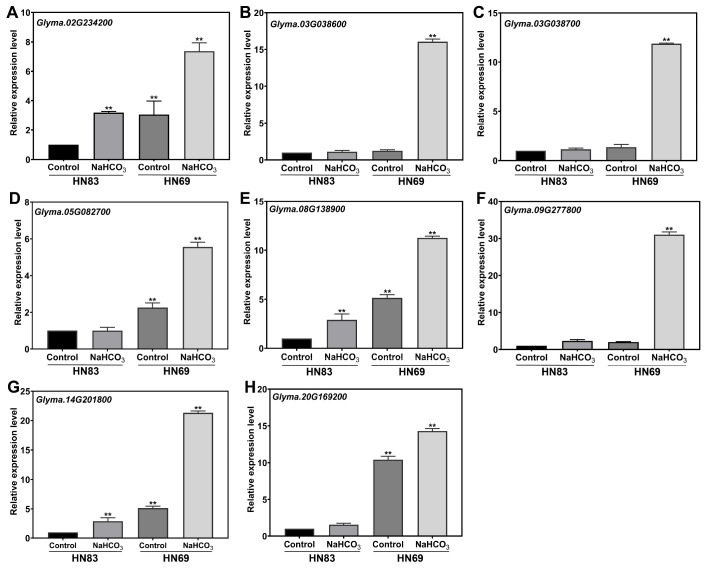
Identification of upregulated differentially expressed genes by qRT-PCR. (**A**–**H**) Whole-plant samples of HN69 and alkali-sensitive HN83 were collected after 6 h alkali treatment and control conditions. *GADPH* was used as an internal control. Data are the means ± SD. ** *p* < 0.01.

**Figure 6:**
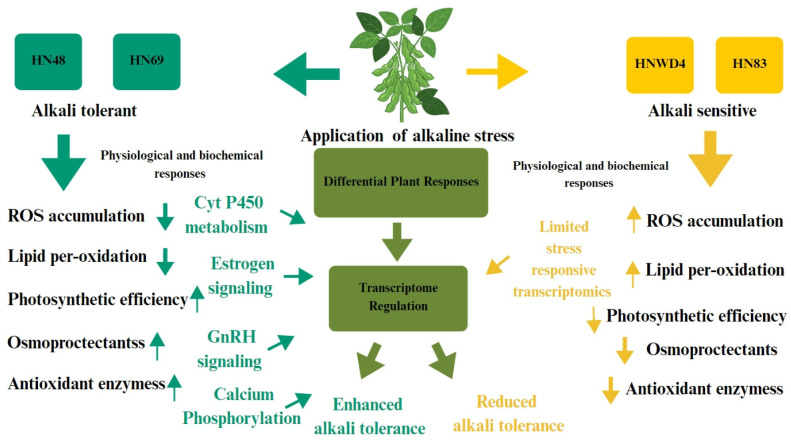
Integrated schematic representation responses underlying alkali stress tolerance in alkali-tolerant and alkali-sensitive varieties in soybean.

To validate the reliability of transcriptome data, eight upregulated peroxidase (POD)-associated genes with high fold changes were selected for qRT-PCR verification. Whole-plant samples of HN69 and the alkali-sensitive HN83 were collected after 6 h of alkali treatment and control conditions, followed by RNA extraction and reverse transcription. As shown in Figure 5, the expression patterns of all eight genes were consistent with the transcriptome results. Notably, the peroxidase genes *Glyma.03G038600* and *Glyma.09G277800* exhibited no significant changes between stress and control conditions in HN83 but showed significant upregulation in HN69, suggesting enhanced antioxidant defense capacity in the tolerant cultivar. An integrated schematic representation of morphological, physiological, biochemical, and transcriptomic responses underlying alkali stress tolerance in soybean is presented in Figure 6.

These results confirm high concordance between qRT-PCR and transcriptome data. The significantly upregulated expression of *Glyma.03G038600* in alkali-stressed HN69 suggests its potential as a candidate gene for further investigation of alkali tolerance mechanisms.

## 3. Discussion

Soil alkalization represents a major abiotic stress factor that severely restricts global crop productivity and poses an escalating threat to food security. Soybean is an essential source of food, vegetable oil, and animal feed. Soil alkalization frequently causes sharp reductions in soybean yield. Therefore, the identification of alkali-tolerant soybean varieties and the elucidation of their physiological and molecular mechanisms of alkali tolerance are still pressing issues in soybean production. In our previous study, we evaluated 99 soybean germplasms from Northeast China and obtained the alkali-tolerant varieties HN48 and HN69, along with the alkali-sensitive varieties HNWD4 and HN83. By integrating multi-stage phenotypic, biochemical, and molecular analyses, we aim to uncover the regulatory mechanisms of alkali tolerance and provide a foundation for breeding resilient soybean varieties for saline–alkali soils.

In the present study, the results showed that the alkali-tolerant varieties HN48 and HN69 displayed better growth morphological parameters, including plant height, root length, fresh weight, and dry weight following exposure to alkaline conditions (Figure 1E–H). Physiological regulation constitutes the core mechanism underlying plants’ adaptation to environmental changes. The metabolism of chlorophyll is highly susceptible to abiotic stress, such as salt, drought, and alkali stresses [17]. We also found that the alkali-sensitive varieties HNWD4 and HN83 exhibited significant chlorophyll decreases compared with the alkali-tolerant varieties HN48 and HN69 (Figure 2A). As a key indicator of chloroplast function, the chlorophyll content also directly determines the photosynthetic efficiency of plant leaves under both normal and stress conditions [18]. We found that the tolerant variety exhibited only a minor reduction in Fv/Fm, maintaining near-normal photosynthetic efficiency (Figure 2B). These findings indicate that alkali-tolerant soybean genotypes mitigate stress-induced damage by maintaining higher chlorophyll content and preserving PSII functionality, thereby enhancing their photosynthetic resilience under alkaline conditions.

Under stress conditions, the plant cells undergo oxidative damage and lipid peroxidation, leading to the accumulation of MDA [19]. In the present study, we showed that the contents of MDA significant were significantly increased in the alkali-sensitive varieties HNWD4 and HN83 (Figure 2C), indicating increased oxidative damage, superoxide anion (O_2_^−^) production, and H_2_O_2_ levels (Figure 2E,F). Therefore, alkali-tolerant varieties mitigate injury through membrane integrity maintenance, osmolyte accumulation, and antioxidant system activation (SOD/CAT/POD) (Figure 3) [20,21].

Transcriptomic analysis revealed divergent gene expression patterns between the alkali-tolerant ‘HN 69’ and the sensitive HN83 under stress. qRT-PCR validation confirmed substantial upregulation of antioxidant enzyme-related genes in HN69. In alkali-tolerant soybean cultivar HN69, GO/KEGG enrichment analysis revealed activation of three pathways: phenylpropanoid biosynthesis eliminates ROS via phenolic compounds [22], MAPK signaling cascade and phytohormone transduction pathway. Key transcription factors were specifically induced, including 22 *ERF* family members (e.g., *GmERF71* enhancing tolerance through *H^+^-ATPase* regulation), 27 NAC members, 31 bHLH members, and 55 MYB members (e.g., GmMYB68 conferring dual salt–alkali resistance). KEGG analysis further identified specific activation of cytochrome P450-mediated drug metabolism and GnRH/estrogen signaling pathways. The enrichment of the estrogen and GnRH signaling pathways in soybeans under alkali stress should be understood in the context of KEGG annotation principles rather than as proof of animal-specific hormonal signaling, even if these pathways are traditionally described in animal systems. Calcium-dependent kinases, MAPK cascades, phosphatidylinositol signaling elements, and cytochrome P450 enzymes are among the conserved molecular components shared by eukaryotes that form the basis of KEGG pathway mapping. Many of these components are essential for plants’ stress perception, signal transduction, and metabolic regulation [23]. Genes associated with the estrogen signaling pathway in plants frequently overlap with redox homeostasis, cytochrome P450-mediated detoxification mechanisms, and secondary metabolite biosynthesis—all of which are known to support abiotic stress tolerance [24]. Therefore, the particular enrichment of these pathways in the alkali-tolerant cultivar HN69 probably indicates the activation of metabolic networks and conserved stress-responsive signaling, which may contribute to cultivar-dependent variations in alkali stress adaption. Similarly, despite variations in upstream ligands, the GnRH signaling pathway contains conserved Ca^2+^-dependent signaling and phosphorylation modules that are functionally relevant to plant hormone crosstalk and stress-responsive transcriptional regulation. These molecular components form a multi-tiered tolerance strategy wherein phenylpropanoid metabolism enhances ROS clearance [25]. MAPK/hormone networks amplify stress signaling, and P450 enzymes potentiate detoxification. This integrated regulatory framework establishes the molecular basis for alkali-tolerant phenotypes [26]. This study employs phenotypically screened alkali-tolerant and sensitive soybean germplasms to elucidate physiological adaptation mechanisms. Integrated transcriptome analysis identifies pivotal tolerance genes [27], establishing a theoretical foundation for molecular breeding of alkali-resistant soybean cultivars [28].

## 4. Conclusions

Taken together, we obtained the alkali-tolerant varieties HN48 and HN69, along with the alkali-sensitive varieties HNWD4 and HN83. By integrating multi-stage phenotypic, biochemical, and molecular analyses, we aim to uncover the regulatory mechanisms of alkali tolerance and provide a foundation for breeding resilient soybean varieties for saline–alkali soils. However, the functions of key regulatory genes identified via transcriptome analysis still require further investigation. Further investigations aim to identify candidate alkali-tolerant genes from alkali-tolerant soybean varieties. Secondly, metabolomic profiling of alkali-tolerant and alkali-sensitive soybean varieties under alkali stress could help reveal differences in their molecular tolerance mechanisms.

## 5. Materials and Methods

### 5.1. Plant Materials

The soybean accessions used in this study were obtained from the Soybean Research Institute, Heilongjiang Academy of Agricultural Sciences. Among these, the alkaline-tolerant cultivars Heinong48 (HN48) and Heinong69 (HN69), along with the alkaline-sensitive cultivars HeinongWangdou4 (HNWD4) and Heinong83 (HN83), were selected to evaluate morphological responses under alkaline stress and to conduct physiological assessments at the seedling stage. For transcriptome analysis, seedlings of HN 69 and HN 83 were specifically chosen to represent tolerant and sensitive genotypes, respectively.

### 5.2. Morphological Assessment of Soybean Under Alkaline Stress

Soybean seeds were surface-sterilized, soaked in distilled water, and germinated in Petri dishes for four days. Uniform seedlings were subsequently transferred to Hoagland’s nutrient solution, placed on floating supports, and cultivated under controlled environmental conditions (25/20 °C day/night temperature and a 16/8 h light/dark photoperiod). After fifteen days of germination and growth, the seedlings were divided into two treatment groups: (1) a control group (CK), maintained in standard Hoagland’s solution, and (2) an alkali stress group, exposed to 30 mM NaHCO_3_. The nutrient solutions were replaced every three days to ensure stable nutrient availability and consistent experimental conditions. Seventy-two hours after treatment, twenty seedlings from each group were randomly selected for morphological evaluation. Root length and shoot height were measured immediately, and the plants were subsequently oven-dried at 65 °C for 12 h to determine dry biomass. For biochemical analyses, approximately 0.1 g of fresh leaf tissue was rapidly frozen in liquid nitrogen and stored at −80 °C until further use.

### 5.3. Physiological Assessment of Soybean Under Alkaline Stress

Chlorophyll content was quantified spectrophotometrically following extraction with acetone by using the method described in [29], while the maximum photochemical efficiency of photosystem II (Fv/Fm) was measured using an Imaging-PAM chlorophyll fluorometer (Walz, Nürnberg, Germany).

### 5.4. Measurement of MDA, H_2_O_2_, O_2_^−^, and Proline Contents

Leaves from both alkali-stressed and control seedlings were collected for biochemical assays following established protocols to assess oxidative stress and osmolyte accumulation. Lipid peroxidation rate was quantified by using the thiobarbituric acid (TBA) reaction method, as described by Heath and Packer [30]. For this purpose, 0.5 g of leaf tissue was homogenized in 5 mL of 10% (*w*/*v*) trichloroacetic acid (TCA) and centrifuged at 11,000 r·min^−1^ for 15 min. The resulting supernatant was mixed with 0.5% (*w*/*v*) TBA prepared in 10% TCA, and the mixture was heated in boiling water for 30 min. After rapid cooling, absorbance was recorded at 532 nm and corrected for nonspecific turbidity at 600 nm. MDA content was expressed as nmol g^−1^ fresh weight. Free proline content was estimated following the ninhydrin–acetic acid method of Bates [31]. According to this method, fresh leaf samples (0.5 g) were homogenized in 3% sulfosalicylic acid and centrifuged at 3000 r·min^−1^ for 10 min. The supernatant was reacted with acid ninhydrin and glacial acetic acid in equal volumes and incubated at 70 °C for 55 min. After cooling, the reaction mixture was extracted with toluene, and absorbance was measured at 520 nm. Proline concentration was calculated using a standard curve of L-proline. H_2_O_2_ concentration was determined according to the method of Velikova [32]. Leaf tissue (0.5 g) was homogenized in 0.1% TCA and centrifuged at 12,000 r·min^−1^ for 22 min at 4 °C. The supernatant was mixed with 10 mM potassium phosphate buffer (pH 7.0) and 1 M potassium iodide (KI). The absorbance was recorded at 390 nm, and H_2_O_2_ concentration was calculated using a standard curve prepared with known concentrations of H_2_O_2_. Furthermore, O^2−^ generation rate was measured following the hydroxylamine oxidation method described by Elstner and Heupel [33]. In this method, fresh samples were incubated in phosphate buffer containing hydroxylamine hydrochloride at room temperature. After extraction with diethyl ether, sulfanilic acid and α-naphthylamine were added sequentially, and the mixture was left to react at 25 °C for 20 min. The absorbance was measured at 530 nm to estimate O^2−^ levels. All biochemical measurements were performed with twenty seedlings from each group per treatment to ensure data accuracy and reproducibility.

### 5.5. Determination of Antioxidant Enzyme Activities

Leaves and roots (0.1 g) from both alkali-stressed and control soybean seedlings were collected to assess antioxidant enzyme activities following standard protocols. Peroxidase (POD) activity was determined using the method of Chance and Maehly [34]. Tissues were homogenized in 50 mM phosphate buffer (pH 7.0) under chilled conditions and centrifuged. The reaction mixture contained guaiacol and H_2_O_2_ in phosphate buffer, and the increase in absorbance due to tetraguaiacol formation was recorded at 470 nm. Superoxide dismutase (SOD) activity was measured according to Giannopolitis and Ries [35], based on the inhibition of nitroblue tetrazolium (NBT) photoreduction. Extracts prepared in 50 mM phosphate buffer (pH 7.8) were centrifuged, and the reaction mixture contained methionine, NBT, riboflavin, EDTA, and phosphate buffer. Absorbance was recorded at 560 nm after illumination. One unit of SOD activity corresponded to 50% inhibition of NBT reduction. Catalase (CAT) activity was determined following Aebi [36]. Samples were homogenized in phosphate buffer (pH 7.0) and centrifuged at 4 °C. The decomposition of H_2_O_2_ was monitored by a decline in absorbance at 240 nm, and CAT activity was expressed as the change in absorbance per minute per gram fresh weight. Ascorbate peroxidase (APX) activity was assayed using the method of Nakano and Asada [37]. Tissues were homogenized in phosphate buffer (pH 7.0) containing 1 mM EDTA and 1% polyvinylpyrrolidone (PVP), followed by centrifugation at 4 °C. The reaction mixture contained phosphate buffer, ascorbic acid, and H_2_O_2_, and the decrease in absorbance at 290 nm due to ascorbate oxidation was recorded.

### 5.6. Stress Treatments, Cultivation of Soybean, and Total RNA Extraction

Soybean seedlings were cultivated under controlled conditions following the procedure of Xu [38], with slight modifications. Uniform seedlings were exposed to either alkali stress (50 mM NaHCO_3_, pH 9.0) or control treatment (distilled water) for 6 h. After treatment, leaf and root samples were collected, immediately frozen in liquid nitrogen, and stored at −80 °C until further analysis. Total RNA was isolated using the Plant Total RNA Extraction Kit (Omega Bio-Tek, Bei Jing, China, R6827-01) according to the manufacturer’s instructions. RNA purity and concentration were assessed using a NanoDrop 2000 spectrophotometer (Thermo Fisher Scientific, Shang Hai, China ) with A260/A280 ratios > 1.8, and integrity was confirmed by 1.5% agarose gel electrophoresis. High-quality RNA samples were sent to AB Life, Inc. (Wuhan, China) for library construction using the NEBNext^®^ Ultra™ RNA Library Prep Kit (New England Biolabs, Ipswich, MA, USA). For transcriptome validation, total RNA was extracted from three biological replicates per treatment using the same protocol.

### 5.7. Analysis of RNA Sequencing Data

After passing quality control, high-quality RNA samples were enriched and reverse-transcribed to synthesize cDNA for library construction following standard Illumina protocols. The resulting cDNA libraries were subjected to high-throughput sequencing, and the clean reads were aligned to the *Glycine max* reference genome obtained from the Phytozome database (https://phytozome-next.jgi.doe.gov/ (accessed on 14 January 2024)) using default alignment parameters to ensure accurate mapping of genomic regions. Gene expression levels were quantified as read counts and normalized for differential expression analysis using DESeq2 [39]. Genes with a false discovery rate (FDR) < 0.05 and an absolute log_2_ fold change (|log_2_FC|) > 1 were considered significantly differentially expressed (upregulated if log_2_FC > 1, downregulated if log_2_FC < −1). Identified DEGs were subjected to Gene Ontology (GO) enrichment analysis using the CARMO web-based platform [40], which categorizes DEGs into molecular function, cellular component, and biological process terms. Enrichment significance was assessed using Fisher’s exact test against the background genome. Functional and pathway enrichment analyses were further performed using the Kyoto Encyclopedia of Genes and Genomes (KEGG) database to identify biological pathways and metabolic processes associated with DEGs.

### 5.8. Differential Gene Expression Analysis

In the differential gene expression analysis, genes with |log2(fold change)| > 1 were identified as DEGs. The DEGs screened from the alkali-tolerant soybean cultivar HN69 and alkali-sensitive cultivar HN83 under both control and 50 mM NaHCO_3_ conditions were visualized. Complementary DNA (cDNA) synthesis was performed using the PrimeScript™ RT Reagent Kit (Takara, Bei Jing, China, RR047A), and quantitative real-time PCR was carried out on a QuantStudio 5 Real-Time PCR System (Applied Biosystems, Shang Hai, China) with SYBR^®^ Premix Ex Taq™ (Takara). Relative gene expression levels were calculated using the 2^−ΔΔCt^ method of Livak and Schmittgen [41], with *GADPH* (*Glyma.06G172700*) as the internal reference gene.

### 5.9. Statistical Analysis

For morphological evaluation and biochemical analyses, the data were analyzed using one-way ANOVA, followed by Duncan’s multiple range test (*p* < 0.05). For qRT-PCR data analysis, the data were analyzed with at least three independent biological replicates. qRT-PCR results were calculated using the 2^−ΔΔCT^ method and Student’s *t*-test. All statistical analyses were conducted using SPSS 21.0 software. Graphical data were visualized using GraphPad Prism 8.0.1.

## Data Availability

Data are contained within the article are available on request from the corresponding author. The data are not publicly available due to privacy and ethical restrictions.

## Supplementary Materials

The following supporting information can be downloaded at https://www.mdpi.com/article/10.3390/plants15020222/s1: Figure S1: GO enrichment bubble plot of differentially expressed genes in HN69. Figure S2: GO enrichment bubble plot of differentially expressed genes in HN83. Figure S3: Bubble map of KEGG enrichment of differentially expressed genes in HN69. Figure S4: Bubble map of KEGG enrichment of differentially expressed genes in HN83. Table S1: Summary of sequencing data. Table S2: Gene-specific primers used in this study.

## Abbreviations

The following abbreviations are used in this manuscript:

HN48	Heinong 48
HN69	Heinong 69
HNWD4	Heinong Wangdou 4
HN83	Heinong 83
SOD	Superoxide dismutase
POD	Peroxidase
CAT	Catalase
MDA	Malondialdehyde
ROS	Reactive oxygen species
qRT-PCR	Quantitative real-time PCR
O_2_^−^	Superoxide anion
