# Supplementary material for: Integrated Evaluation of Alkaline Tolerance in Soybean: Linking Germplasm Screening with Physiological, Biochemical, and Molecular Responses
Source: Plants (Basel). 2026 Jan 10;15(2):222. doi: 10.3390/plants15020222 (PMC12845270; doi:10.3390/plants15020222)
Supplement: Supplementary file 1 [file plants-15-00222-s001.zip › Table S1.pdf]

Supplementary Table S1. Summary of sequencing data

Samples	Total Raw Reads (M)	Total Clean Reads (M)	Total Clean Bases(Gb)	Clean Reads Ratio(%)	Q20%	Q30%	Total Mapping(%)
Control-HN69-1	47927498	45992410	95.96	0.04	98.49	95.01	43.97
Control-HN69-2	53282386	51322754	96.32	0.04	98.47	94.41	44.03
Control-HN69-3	52223122	50195804	96.12	0.04	98.68	95.16	44.19
Control-HN83-1	54327190	52310824	96.29	0.04	98.67	95.15	43.92
Control-HN83-2	56974036	55377786	97.20	0.04	98.87	95.85	44.00
Control-HN83-3	50663578	48963796	96.64	0.04	98.6	94.88	43.96
Alkaline-HN69-1	41639360	39936872	95.91	0.04	98.61	94.98	43.91
Alkaline-HN69-2	51734018	49662300	96.00	0.04	98.82	95.77	44.15
Alkaline-HN69-3	50902592	49109508	96.48	0.04	98.79	95.63	43.97
Alkaline-HN83-1	51893912	50138764	96.62	0.04	98.81	95.72	43.87
Alkaline-HN83-2	46459140	44630204	96.06	0.04	98.66	95.15	43.65
Alkaline-HN83-3	49501546	47196948	95.34	0.04	98.53	94.72	43.46
