# Supplementary material for: Integrated Evaluation of Alkaline Tolerance in Soybean: Linking Germplasm Screening with Physiological, Biochemical, and Molecular Responses
Source: Plants (Basel). 2026 Jan 10;15(2):222. doi: 10.3390/plants15020222 (PMC12845270; doi:10.3390/plants15020222)
Supplement: Supplementary file 1 [file plants-15-00222-s001.zip › Table S2.pdf]

Supplementary Table S2 Gene-specific primers used in this study

Gene ID	Primer sequence (5′-3′)
GmGADPH	Forward: GACTGGTATGGCATTCCGTGT
	Reverse: GCCCTCTAGTTCCTCCTTGA
Glyma.03G038600	Forward: GCAGATATATTGGCTGTTGCC
	Reverse: CTTTGCTTGCTGTGGTGGA
Glyma.09G277800	Forward: CAAGTGCTTTTCAATGGAGGA
	Reverse: GCCAGTAAGTGGGCTAATGTTT
Glyma.03G038700	Forward: TATTGACTGTTGCCGCTCG
	Reverse: CATGGCTCTTGAAGTTGCTTATG
Glyma.20G169200	Forward: GGCTTGCTTCGCCTTCACT
	Reverse: CGACTCTTCCACAACTCTTTTCTA
Glyma.14G201800	Forward: ATTCCACGACTGGCTTTTTCA
	Reverse: CATTGTTGTCCTTTGGTGTTCC
Glyma.02G234200	Forward: CATCTATCGTTCGCCTCTTCTT
	Reverse: CTCCGGGGCATACTGTCTC
Glyma.05G082700	Forward: AATACTCCCACATCCATAGCCA
	Reverse: CGACCCAGAAGTCATAACCGT
Glyma.08G138900	Forward: CATTTGTGGTGAAAAACCTGTT
	Reverse: GGATGTACCCTTGGCGTGC
