# Supplementary figures and images for: Integrated Evaluation of Alkaline Tolerance in Soybean: Linking Germplasm Screening with Physiological, Biochemical, and Molecular Responses
Source: Plants (Basel). 2026 Jan 10;15(2):222. doi: 10.3390/plants15020222 (PMC12845270; doi:10.3390/plants15020222)

**Supplementary Figure S1.** GO enrichment bubble plot of differentially expressed genes in HN69.

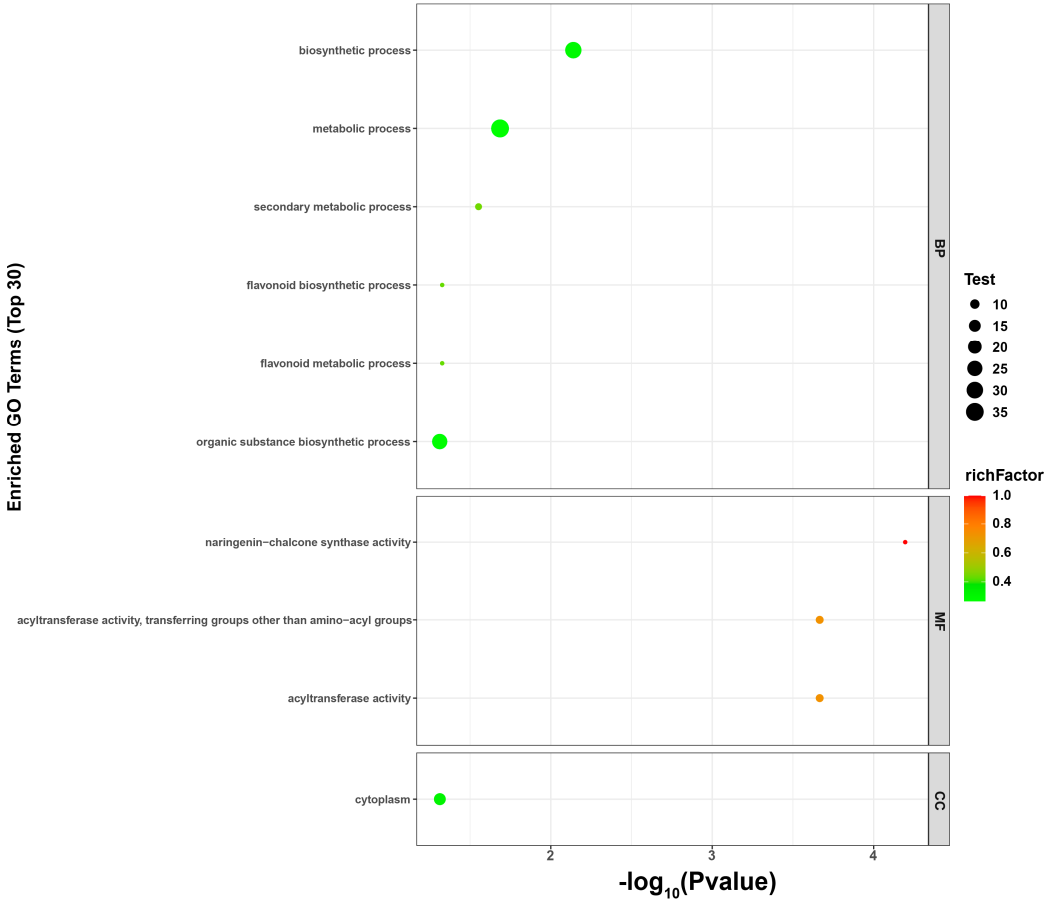

Supplement: Supplementary file 1 [file plants-15-00222-s001.zip › Figure S1.pdf]

Supplementary Figure S2. GO enrichment bubble plot of differentially expressed genes in HN83.

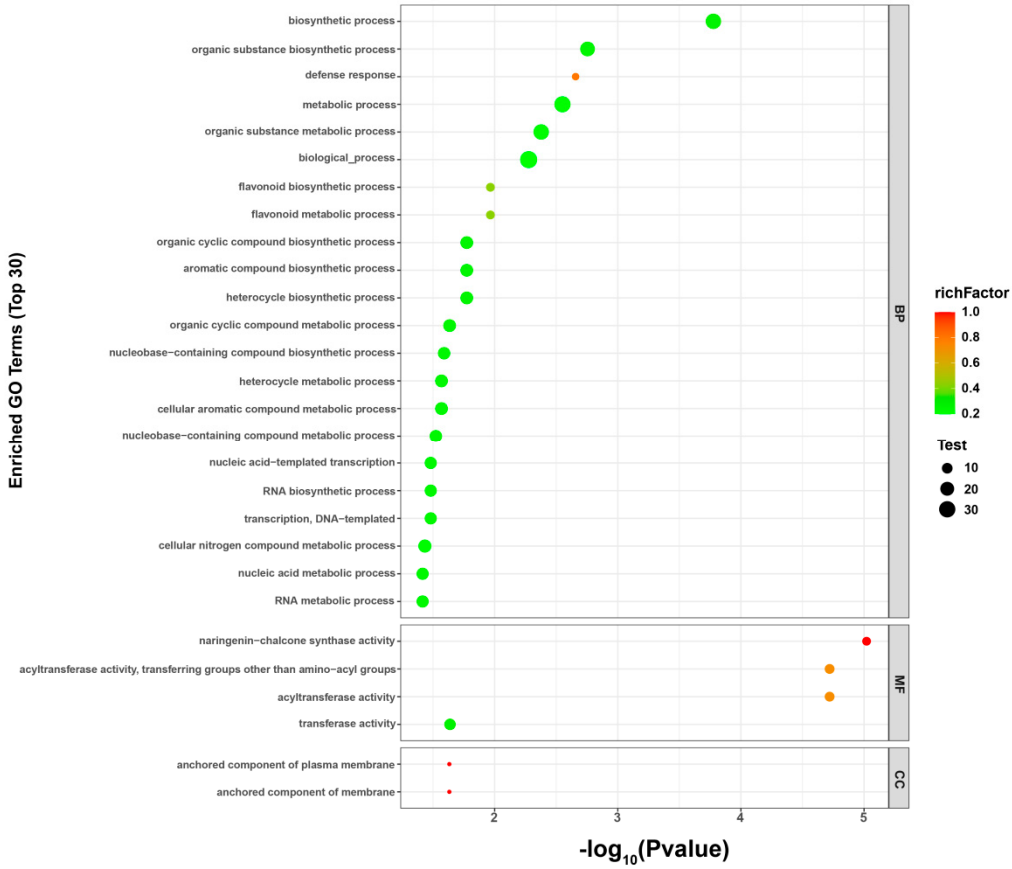

Supplement: Supplementary file 1 [file plants-15-00222-s001.zip › Figure S2.pdf]

**Supplementary Figure S3.** Bubble map of KEGG enrichment of differentially expressed genes in HN69.

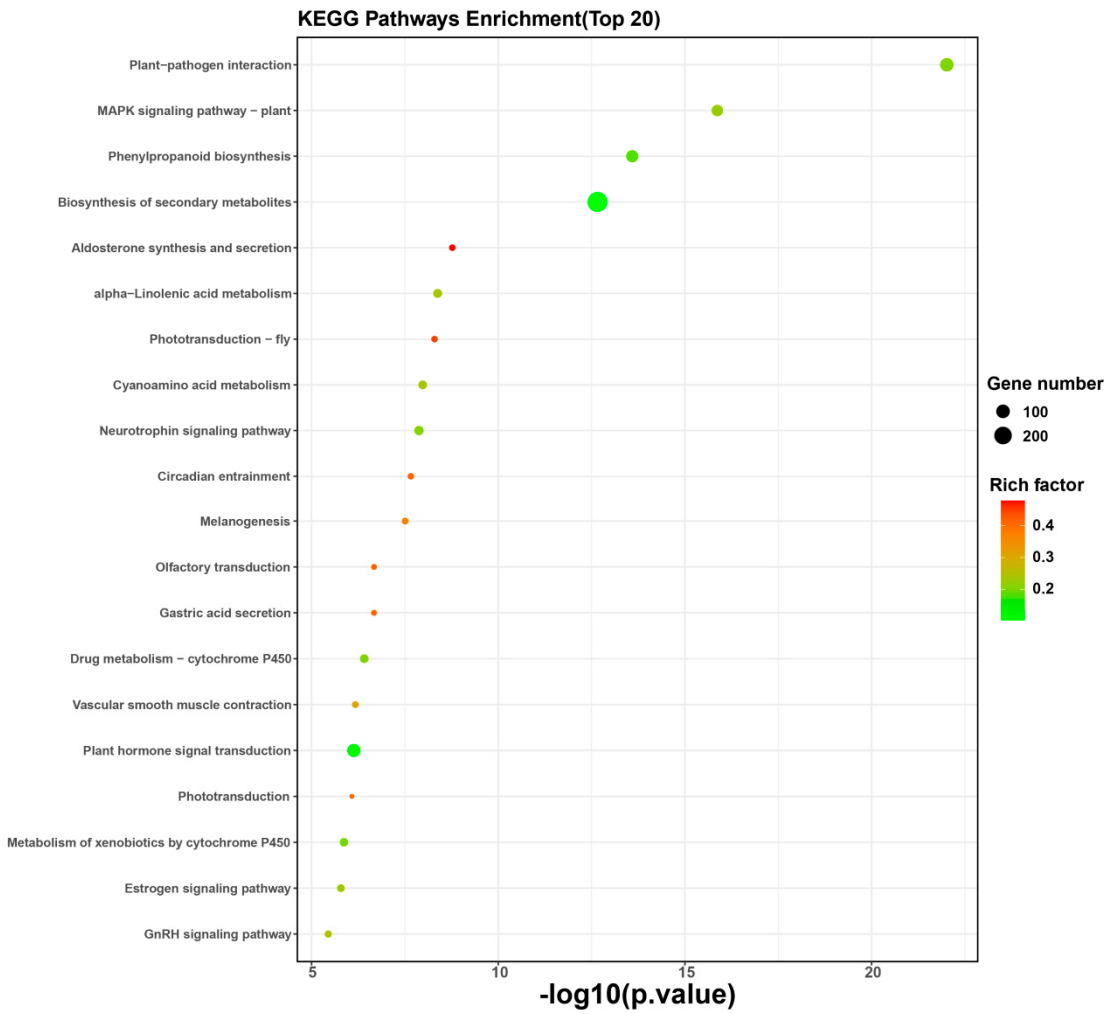

Supplement: Supplementary file 1 [file plants-15-00222-s001.zip › Figure S3.pdf]

**Supplementary Figure S4.** Bubble map of KEGG enrichment of differentially expressed genes in HN83.

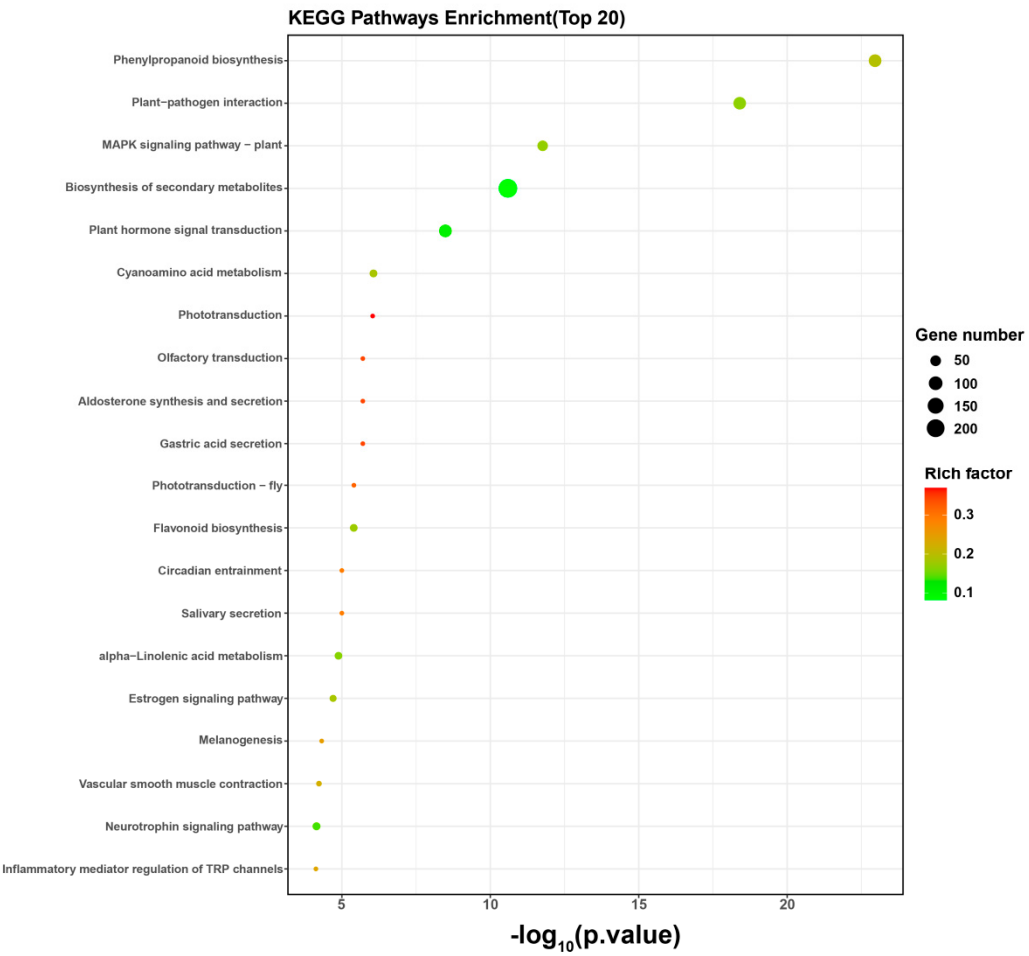

Supplement: Supplementary file 1 [file plants-15-00222-s001.zip › Figure S4.pdf]
